# Biotransformation of ginsenoside Rb1 via the gypenoside pathway by human gut bacteria

**DOI:** 10.1186/1749-8546-8-22

**Published:** 2013-11-23

**Authors:** Hong Shen, Weng-Im Leung, Jian-Qing Ruan, Song-Lin Li, Jacky Pui-Cheong Lei, Yi-Tao Wang, Ru Yan

**Affiliations:** 1 State Key Laboratory of Quality Research in Chinese Medicine, Institute of Chinese Medical Sciences, University of Macau, Macao, China; 2 Department of Pharmaceutical Analysis & Metabolomics, Jiangsu Provincial Academy of Traditional Chinese Medicine, Nanjing, Jiangsu, China; 3 Clinical Laboratory, Kiang Wu Hospital, Estrada do Repouso, Macao, China

## Abstract

**Background:**

Bacterial conversion of ginsenosides is crucial for the health-promoting effects of ginsenosides. Previous studies on the biotransformation of ginsenoside Rb1 (Rb1) by gut bacteria have focused on the ginsenoside Rd (Rd) pathway (Rb1 → Rd → ginsenoside F2 (F2) → compound K (Cpd K)). This study aims to examine the gypenoside pathway in human gut bacteria *in vitro*.

**Methods:**

The metabolic pathways of ginsenoside Rb1 and its metabolites ginsenoside Rd and gypenoside XVII in human gut bacteria were investigated by incubating the compounds anaerobically with pooled or individual gut bacteria samples from healthy volunteers. Ginsenoside Rb1, the metabolites generated by human gut bacteria, and degraded products in simulated gastric fluid (SGF) were qualitatively analyzed using an LC/MSD Trap system in the negative ion mode and quantitatively determined by HPLC-UV analysis.

**Results:**

When incubated anaerobically with pooled gut bacteria, Rb1 generated five metabolites, namely Rd, F2, Cpd K, and the rare gypenosides XVII (G-XVII) and LXXV (G-LXXV). The gypenoside pathway (Rb1 → G-XVII → G-LXXV → Cpd K) was rapid, intermediate, and minor, and finally converted Rb1 to Cpd K *via* G-XVII → F2 (major)/G-LXXV (minor). Both the Rd and gypenoside pathways exhibited great inter-individual variations in age-and sex-independent manners (*P* > 0.05). Rb1 was highly acid-labile and degraded rapidly to form F2, ginsenoside Rg3, ginsenoside Rh2, and Cpd K, but did not generate the gypenosides in SGF. The formation of the gypenosides might be explained by the involvement of a gut bacteria-mediated enzymatic process.

**Conclusions:**

Rb1 was metabolized to G-XVII, F2 (major) or G-LXXL (minor), and finally Cpd K by human gut bacteria *in vitro*.

## Background

Ginsenosides are the major bioactive components in *Panax*, including *Panax ginseng*, *Panax quinquefolium*, and *Panax notoginseng *[1], which are the most popular herbs used for medicinal and nutritional purposes in China, Japan, Korea, and some Western countries. Two dammarane-type triterpene *O*-glycosides, namely 20(*S*)-protopanaxadiol (PPD) and 20(*S*)-protopanaxatriol, are the major ginsenosides in different parts of *Panax*, including the flower bud, berry, crown, rootlet, side root, seed bud, seed, stem, and leaf [2,3], and their differently processed herbal products [4]. As one of the most important PPD-type ginsenosides, ginsenoside Rb1 (Rb1) has exhibited various pharmacological activities, including neuroprotective, antitumor, cardiovascular-protective, and anti-aging effects, in many *in vitro* and *in vivo* models [5-7].

The intestinal absorption of glycosides in plants or foods is poor, while their metabolites generated by intestinal microflora are more permeable and bioactive [8-11]. The oral bioavailability of PPD-type ginsenosides is generally low (<10%) [12]. The bacterial metabolites are the main forms transported across the epithelial membrane and are most likely to be the real *in vivo* active forms [13]. This finding has led to research on microbial metabolism and the pharmacological activities of the resultant metabolites of ginsenosides including Rb1 [14-16]. Hasegawa *et al. *[17] proposed metabolism of Rb1 *via* the ginsenoside Rd (Rd) pathway by human intestinal bacteria *in vitro*, which was initiated at the C-20 glucose (Rb1 → Rd → ginsenoside F2 (F2) → Compound K (Cpd K)), and the gypenoside XVII (G-XVII) pathway, which was initiated by removal of the C-3 glucose (Rb1 → G-XVII → gypenoside LXXV (G-LXXV) → Cpd K). However, the gypenoside pathway of Rb1 was only speculated from the results of TLC, and the data were not provided by the authors [18,19].

The formation of G-XVII from Rb1 has been investigated in several metabolic studies with individual fungal strains [20-22]. Moreover, G-XVII was detected *in vivo* in rats receiving oral administration of Rb1 [23,24]. Although there are no reports on the formation of G-LXXV in rats, the presence of G-XVII suggests the existence of both the gypenoside and Rd pathways of Rb1 metabolism in rats. In plasma and urine samples from healthy subjects who received oral administration of a ginseng extract, only Rd and Cpd K were detected [25,26]. The gypenoside pathway of Rb1 metabolism in the human body remains to be confirmed.

Ginsenoside Rd and Cpd K have been shown to possess potent pharmacological activities [27], contributing to the beneficial effects of Rb1. The available, albeit limited, reports on the bioactivities of gypenosides have described that the gypenosides from *Gynostemma pentaphyllum* have anticancer, antioxidative, and antihyperlipidemic effects [28,29]. It is important to know whether gypenosides can be formed from ginsenoside Rb1 by bacterial biotransformation in the human gut lumen.

Gypenosides could be generated from ginsenoside Rb1 *via* a non-enzymatic process *in vivo*, such as degradation in the acidic environment of the stomach [30], other than biotransformation by gut bacteria. Several studies have reported the degradation of several ginsenosides, including Rb1, in acidic environment [31,32]. However, the aspect of whether Rb1 is degraded to form gypenosides remains unclear.

This study aims to examine the gypenoside pathway in human gut bacteria *in vitro*. The biotransformation of Rb1 by human gut bacteria was characterized by high-performance liquid chromatography with a UV detector (HPLC-UV) and HPLC-mass spectrometry (MS) analyses. The gypenoside pathway of Rb1 was investigated and compared with the Rd pathway by incubating Rb1, Rd, and G-XVII separately with pooled human gut bacterial samples. The inter-individual variations in the G-XVII and Rd pathways were also characterized with gut bacterial samples from 58 individuals of both sexes and different ages. The stabilities of Rb1 and Rd in simulated gastric fluid (SGF) were examined to rule out the generation of these gypenosides under the acidic gastric environment.

## Methods

### Chemicals, reagents, and materials

Ginsenoside Rb1, Rd, F2, Rg3, Cpd K, Rh2, G-XVII, and Rg1 were supplied by Chengdu Must Biotechnology Co. Ltd. (Chengdu, China). The purity was >98% (HPLC-UV). The BBL™ Brain Heart Infusion (BHI) medium, GasPak™ EZ anaerobe container system with an indicator, and GasPak™ EZ large incubation container were purchased from Becton Dickinson (Franklin Lakes, NJ, USA). L-cystine was purchased from Research Organics Inc. (Cleveland, OH, USA). Dimethyl sulfoxide (DMSO), bovine hemin, and vitamin K1 were supplied by Sigma-Aldrich (St. Louis, MO, USA). Methanol, 1-butanol, and acetonitrile were HPLC-grade and purchased from Merck (Darmstadt, Germany). Dulbecco’s phosphate-buffered saline (PBS) was provided by Life Technologies (Carlsbad, CA, USA). Hydrochloric acid and sodium carbonate (analytical grade) were purchased from Sigma-Aldrich (St. Louis, MO, USA). Deionized water was prepared in-house using a Milli-Q purification system (Millipore, Bedford, MA, USA).

### Instrumentation and analytical conditions

Chromatographic separation was performed on an Agilent 1200 Series HPLC apparatus (Agilent Technologies, Santa Clara, CA, USA) equipped with a vacuum degasser, a binary pump, and an autosampler, and connected to a diode array detector (DAD). Samples were loaded onto an Alltima C18 column (250 × 4.6 mm, 5 μm). The column temperature was maintained at 25°C. The mobile phase consisted of water (A) and acetonitrile (B) and flowed at 1.5 mL/min. The following gradient elution program was used for samples obtained from bacterial incubations: 0-10 min, 19-20% B; 10-28 min, 20-30% B; 28-40 min, 30-60% B; 40-48 min, 60-100% B. The elution program was modified when the incubations of ginsenosides with SGF were analyzed as follows: 0-10 min, 19-20% B; 10-28 min, 20-30% B; 28-35 min, 30-60% B; 35-45 min, 60-100% B. Rb1 and its bacterial metabolites were monitored at 203.4 nm and their UV absorbance was recorded over 200-400 nm. The injection volume was 5 μL.

MS analysis was performed on an liquid chromatograph/mass selective detector (LC/MSD) Trap system (Agilent Technologies, Palo Alto, CA, USA) equipped with an ion-trap mass spectrometer with an electrospray ionization (ESI) interface. Agilent ChemStation for LC 3D System software (Rev. B. 01. 03-SR2) (Agilent Technologies, Santa Clara, CA, USA) was used for instrument control, data acquisition, and data management. Except for replacement of the water with 50 mM ammonium acetate in water, the mobile phase composition and gradient elution program were the same as those for the above HPLC analysis method for human gut bacteria samples. The mass spectrometer was operated in the negative ion mode with the following conditions: drying gas (N_2_), 8 L/min; temperature, 325°C; nebulizer pressure, 30 psi; scan range, *m/z* 100-1400. The ESI-MS/MS conditions were as follows: negative ion mode; separation width, 4; fragment amplification, 1.0 V; scan range, *m/z* 100-1400.

### Preparation of human gut bacteria

The medium was prepared according to our previous report [33]. Briefly, 100 mL of autoclaved BHI medium (3.7 g/100 mL) was supplemented with 0.05 mg of vitamin K1, 0.5 mg of bovine hemin, and 50 mg of L-cystine. The fecal samples (healthy Chinese, 18-92 years old) were provided by Kiang Wu Hospital and the experimental protocol is approved by Kiang Wu Hospital and University of Macau before conduct. Human gut bacteria suspensions were prepared at 4°C according to our previous report [33] with minor modifications. Fecal samples were freshly collected and pooled at 1 g each for identification of Rb1 metabolic pathways, or individually processed at 5 g each for characterization of individual variations in Rb1 metabolism. The individual or pooled fecal samples were mixed with 20 mL of BHI medium and the resulting suspensions were centrifuged at 200 × *g* for 5 min. The supernatants were then centrifuged at 5000 × *g* for 30 min. The human gut bacteria suspensions were obtained by resuspension of the bacterial precipitates in 5 mL of BHI medium.

### Biotransformation of Rb1 by human gut bacteria

Biotransformation assays of Rb1 were conducted with pooled or individual human gut bacteria samples to examine the existence of the gypenoside pathway and compare it with the Rd pathway. A high concentration of Rb1 was used to capture the minor formation and quantitative variations in the metabolites formed from the minor gypenoside pathway based on the great individual variations in gut bacteria compositions and metabolic capabilities [34]. The anaerobic incubation system contained 250 μL of gut bacteria suspension, 100 μL of Rb1 in DMSO (final concentration, 100 mM Rb1), and 4.75 mL of BHI medium. The incubation system was maintained in an anaerobic state at 37°C using the GasPak™ EZ anaerobe container system.

The metabolic pathway and time-course of Rb1 metabolism by human gut bacteria were characterized in five independent experiments using pooled samples. In each experiment, the reaction of Rb1 with a human gut bacteria suspension was conducted in duplicate. Control reactions were conducted in parallel by adding 250 μL of PBS (pH 7.0) instead of gut bacteria suspension. The reactions were terminated at 0, 2, 4, 12, 18, 24, 36, and 48 h, respectively, by adding 15 mL of 1-butanol followed by immediate centrifugation at 5000 × *g* for 30 min to remove the bacteria. The resulting supernatants were mixed with 100 μL of ginsenoside Rg1 (final concentration, 100 mM) as an internal standard (IS), and each mixture was extracted twice with water-saturated 1-butanol. The organic layers were combined and dried by rotary evaporation (BUCHI, Flavil, Switzerland). The residue was reconstituted in 0.2 mL of methanol, and an aliquot (5 μL) was subjected to HPLC-UV or HPLC-MS analysis.

Inter-individual variations in the gypenoside and Rd pathways were investigated by incubating Rb1 with gut bacteria suspensions from different individuals under the same conditions described above, and the incubated samples collected at 18 h were processed as described above. Samples were analyzed by HPLC-UV, and the peak area ratio data of each analyte were collected for comparison.

### Biotransformation of Rd and G-XVII by human gut bacteria

The metabolism of the two positional isomers Rd and G-XVII by human gut bacteria was investigated by incubation with the same human gut bacteria sample in parallel to identify the pathways between Rd/G-XVII and Cpd K. The anaerobic incubation system contained 20 μL of gut bacteria suspension, 10 μL of ginsenoside Rd or G-XVII (final concentration, 100 μM Rd or G-XVII), and 170 μL of BHI medium. The concentrations of Rd and G-XVII were determined based on the high amounts of all metabolites determined from a preliminary study. Each reaction was conducted in triplicate. Control reactions without gut bacteria were conducted in parallel.

Reactions were terminated at 0, 2, 4, 6, 8, 12, 18, 24, 36, and 48 h, respectively, by adding 1.2 mL of 1-butanol followed by centrifugation at 5000 × *g* for 30 min to remove the bacteria. After adding 10 μL of ginsenoside Rg1 (final concentration, 100 μM Rg1) as an internal standard, the resulting mixture was processed in the same manner described above before being subjected to HPLC-UV analysis.

### Stabilities of Rb1 and Rd in SGF

SGF was prepared by diluting 3.84 mL of hydrochloric acid to 1000 mL with deionized water (pH 1.2) according to the Chinese Pharmacopoeia [35]. Ten microliters of ginsenoside Rb1 or Rd in DMSO (final concentration, 71.4 μM) was mixed with 200 μL of SGF, and kept at 37°C for different time intervals (0, 20, 40, 60, 90, 150, 180 and 300 min) before adding 70 μL of 0.1 mol/L Na_2_CO_3_ to examine the acidic stability. Each reaction was conducted in triplicate. The mixture was shaken for 1 min by vortexing and centrifuged at 14,000 × *g* for 5 min. The upper layer was decanted and an aliquot (20 μL) was subjected to HPLC-UV analysis.

### Calibration curves of Rb1 and its metabolites Rd, F2, G-XVII, and Cpd K in human gut bacteria

Serial concentrations of Rb1, Rd, G-XVII, F2, and Cpd K were prepared in DMSO, and mixed with a pooled human gut bacteria suspension and BHI medium as described in the section entitled *Biotransformation of Rb1 by Human Gut Bacteria*. The resulting mixtures were immediately extracted with 1-butanol and centrifuged (5000 × *g*, 30 min). The organic layers were processed in the same manner described above and then subjected to HPLC-UV analysis. Calibration curves were constructed by plotting the peak area ratio of each analyte to the IS (Y) as a function of the analyte concentration (X) in the reaction system.

For measurements of the intra-day precision and accuracy, three concentration levels for each standard (low, medium, and high) were prepared as quality control (QC) samples and analyzed on the same day. The inter-day precision and accuracy were determined with the same three QC samples on three consecutive days.

### Calibration curves of ginsenosides in SGF

A mixed stock solution of Rb1, Rd, G-XVII, F2, Rg3, Cpd K, and Rh2 was prepared in DMSO. A series of working solutions were prepared with DMSO using dilution factors of 2.5, 5, 10, 20, 40, and 80 from the stock solution. An aliquot (10 μL) of each working solution was spiked with 200 μL of SGF and processed with 70 μL of 0.1 mol/L Na_2_CO_3_ as described above. The sample (20 μL) was injected into the HPLC instrument. A calibration curve was obtained by plotting the peak area of each analyte (Y) against its concentration in SGF (X).

### Data analysis

Data were presented as the means ± standard deviation (SD) of triplicate analyses (experiments with pooled human gut bacteria or SGF) or individual measurements (experiments on inter-individual variations). Sex and age differences in ginsenoside Rb1 metabolism were compared by Student’s *t-*test. Values of *P* < 0.05 were considered statistically significant.

## Results

### Metabolism of ginsenoside Rb1 by pooled human gut bacteria

**Figure 1:**
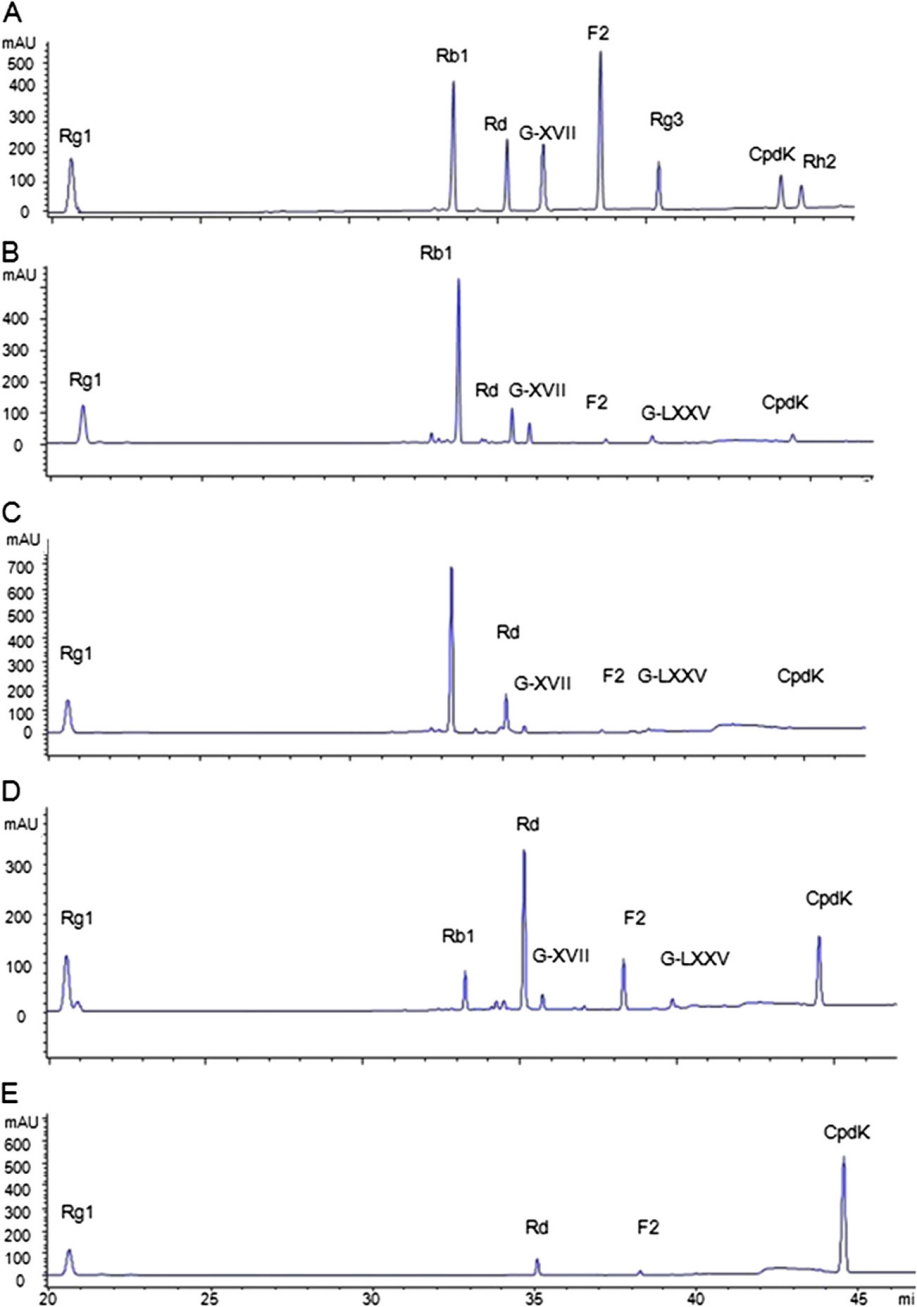
**HPLC-UV chromatograms of (A) mixed ginsenoside standards, and incubated samples of Rb1 with (B) pooled gut bacteria and intestinal bacteria from (C) a 65-year-old male, (D) a 44-year-old male, and (E) a 54-year-old female.** M1, ginsenoside Rd; M2, gypenoside XVII; M3, ginsenoside F2; M4, gypenoside LXXV; M5, compound K.

Fecal samples from 50 healthy volunteers (25 males and 25 females, range, 45.7-73.7 years) were collected and pooled at 10 individuals each to prepare five human gut bacteria pools. When Rb1 was incubated with pooled human gut bacteria, five peaks (M1-M5) were observed at 35.1, 35.6, 38.2, 39.7, and 44.4 min, respectively, which were absent in control reactions (Figure 1). Under the developed analytical conditions, Rb1 and its metabolites were well-separated from the endogenous interferences of the *in vitro* anaerobic incubation system. The retention times and characteristic CID fragment ions of the ginsenoside standards and Rb1 metabolites are summarized in Table 1.

**Table 1:** HPLC-ESI-MS and corresponding CID data of ginsenoside standards and the metabolites of ginsenoside Rb1 by human gut bacteria

**Retention time (min)**	**MS ( *m/z *)**			**CID ( *m/z *)**	**Identity**
	**[M-H]**^**-**^	**[M + Cl]**^**-**^	**[M + AcO]**^**-**^		
20.9	799	835	859	637[M-H-Glc]-, 619[M-H-Glc-H_2_O]-, 475[M-H-2Glc]-	Ginsenoside Rg1 (IS)
33.2	1107	1143	-	945[M-H-Glc]-, 783[M-H-2Glc]-, 621[M-H-3Glc]-	Ginsenoside Rb1
35.1	945	981	-	783[M-H-Glc]-, 621[M-H-2Glc]-,	Ginsenoside Rd (M1)
35.6	945	981	-	783[M-H-Glc]-, 621[M-H-2Glc]-,	Gypenoside XVII (M2)
38.2	783	819	843	621[M-H-Glc]-, 459[M-H-2Glc]-	Ginsenoside F2 (M3)
39.7	783	819	843	621[M-H-Glc]^-^, 459[M-H-2Glc]^-^	Gypenoside LXXV (M4)
40.3	783	819	-	621[M-H-Glc]-, 459[M-H-2Glc]-	Ginsenoside Rg3
45.4	621	657	681	459[M-H-Glc]-	Ginsenoside Rh2
44.4	621	657	681	459[M-H-Glc]-	Compound K (M5)

Rb1 was eluted at 33.2 min and showed its pseudo-molecular ion at *m/z* 1107 ([M-H]^-^) and chloride adduct ion at *m/z* 1143 ([M + Cl]^-^), corresponding to a molecular weight of 1108 Daltons. The fragment ions at *m/z* 945 ([M-H_2_O-H]^-^), *m/z* 783 ([M-2H_2_O-H]^-^), and *m/z* 621 ([M-3H_2_O-H]^-^) in the CID spectrum corresponded to loss of one, two, and three glucose molecules from Rb1, respectively.

The retention times and mass spectral profiles of M1 (35.1 min), M3 (38.2 min), and M5 (44.4 min) were identical to those of Rd, F2, and Cpd K, respectively. The retention time and mass spectral profile of M2 (35.6 min) were identical to those of G-XVII, which was formed from Rb1 by hydrolytic removal of one glucose molecule from the C-3 position of Rb1. Thus, M1–M3 and M5 were unambiguously identified as ginsenosides Rd, G-XVII, F2, and Cpd K, respectively.

M4 was eluted at 39.7 min. The MS spectral profile of M4 was similar to those of M3 (ginsenoside F2) and ginsenoside Rg3 (Table 1), supporting that M4 contained a ginsenoside glycoside with two glucoses. However, the retention time of M4 was different from that of either ginsenoside Rg3 (40.3 min, with two glucoses at the C-3 position) or F2 (38.2 min, with one glucose at the C-3 position and another at the C-20 position) (Figure 1). Thus, M4 was tentatively identified as G-LXXV, a metabolite formed from Rb1 with two glucose molecules remaining at the C-20 position.

Taken together, Rb1 biotransformation by human gut bacteria *in vitro* resulted in the formations of gypenosides as well as metabolites of the Rd pathway.

### Time-course of Rb1 metabolism by human gut bacteria

The calibration curves of Rb1, Rd, F2, G-XVII, and Cpd K in the *in vitro* anaerobic incubation system showed good linearity (*r*^*2*^ > 0.998, *P* < 0.001) over the concentration ranges tested (Table 2). The overall intra-day and inter-day variations were less than 6%, and recovery was higher than 85% (except for ~60% for 10 μM Cpd K) (Additional file 1: Table S1).

**Table 2:** **Calibration curves of ginsenosides in simulated gastric fluid and an *in vitro *** **human gut bacteria incubation system**

**Matrix**	**Analyte**	**Calibration curve (μΜ)**	**r**^**2**^	**Concentration range (μΜ)**
Simulated gastric fluid	Rb1	Y = 3.504X + 6.912	0.9999	2.08-167.00
	Rd	Y = 3.796X-3.843	0.9999	11.30-3618.00
	G-XVII	Y = 3.549X-3.430	0.9999	5.21-1666.67
	F2	Y = 3.339X-1.033	0.9999	5.21-1666.67
	Rg3	Y = 3.377X + 10.180	0.9998	1.85-148.30
	Cpd K	Y = 3.807X + 0.303	0.9999	5.21-1666.67
	Rh2	Y = 3.644X + 6.880	0.9999	5.21-1666.67
Human gut bacteria	Rb1	Y = 6.1209X + 0.094	0.9986	50.00-800.00
	Rd	Y = 7.3891X + 4E-0.5	0.9998	31.25-500.00
	G XVII	Y = 6.1571X + 0.062	0.9999	30.00-200.00
	F2	Y = 6.7242X + 0.003	0.9999	16.25-260.00
	Cpd K	Y = 6.7841X + 0.042	0.9945	5.16-165.00

**Figure 2:**
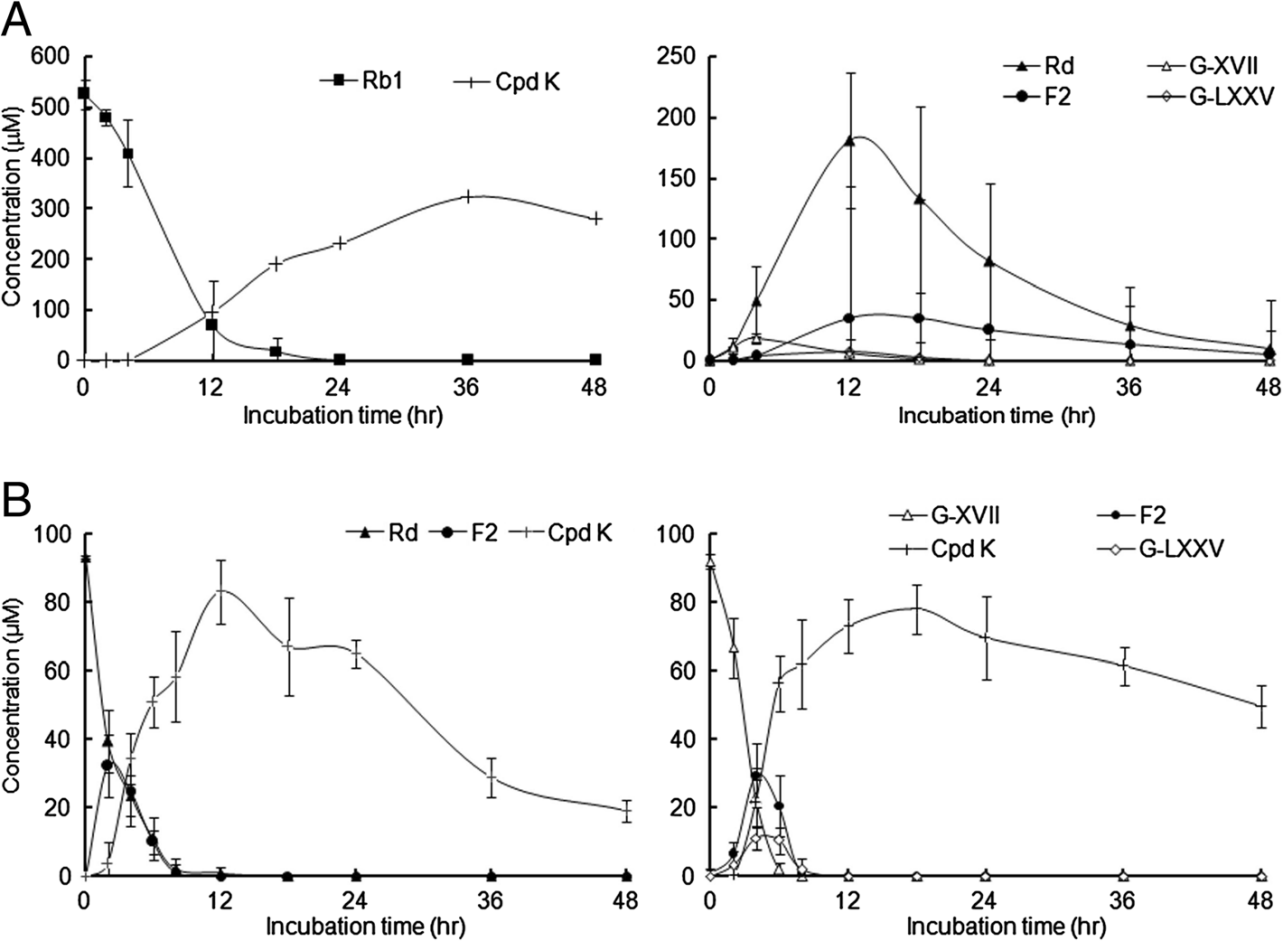
Time-courses of the elimination and metabolite formations of (A) ginsenoside Rb1, and (B) Rd (solid line) and G-XVII (dotted line) in human gut bacteria.

When Rb1 was incubated with the pooled human gut bacteria (Figure 2A), Rb1 was rapidly eliminated by the human gut bacteria and less than 20% of the Rb1 remained intact at 12 h. The metabolites from the Rd pathway, Rd and F2, appeared in the incubations in turn, with Rd reaching its maximum at 12 h (representing ~50% of the initial Rb1) followed by the maximum of F2 at relatively low levels (representing ~7% of the initial Rb1). The amount of Cpd K peaked at 36 h. Compared with their respective positional isomers Rd and F2, G-XVII and G-LXXV formed from the gypenoside pathway reached their maximum levels more rapidly (T_max_: 4 and 8-12 h, respectively). Because the positional isomers Rd and G-XVII showed similar UV responses, as evidenced by the very close constant coefficients (Table 2), we assumed that F2 and G-LXXV, the positional isomers formed by hydrolytic removal of two glucoses from C-3 and C-20 (F2) and C-3 (G-LXXV), would also have similar UV responses. Thus, the levels of the positional isomers F2 and G-LXXV were calculated using the calibration curve of F2. Both gypenosides were formed at relatively lower levels than their positional isomers. The maximum level of G-XVII was about 10% of Rd, while that of G-LXXV was half of the peak concentration of F2. Moreover, both gypenosides had disappeared from the incubation system within 24 h, long before Cpd K reached its maximum level at 36 h, while Rd and F2 were still detectable at 48 h. Thus, the gypenoside pathway should be the minor pathway of Rb1 biotransformation by human gut bacteria.

### Biotransformation of Rd and G-XVII by human gut bacteria

Rd and G-XVII were depleted within 8 h by human gut bacteria, and generated F2 and Cpd K when incubated separately. The generations of F2 and Cpd K from Rd peaked at 2 and 12 h, respectively, while those generated from G-XVII reached their maximum levels at 4 and 18 h, respectively. The maximum levels of F2 and Cpd K formed from Rd were comparable to those from G-XVII (about 30 and 80 μM, reflecting 30% and 80% of the original amounts of the respective parent compounds) (Figure 2B).

G-LXXV was also generated by human gut bacteria as evidenced by the retention time and mass spectral profile. F2 presented as a major metabolite and the C_max_ was two times higher that of G-LXXV (Figure 2B). Therefore, both the G-XVII → F2 and G-XVII → G-LXXV pathways of G-XVII metabolism existed in the human gut bacteria, with the former as the major pathway *in vitro*.

### Rb1 biotransformation by individual human gut bacterial samples

**Figure 3:**
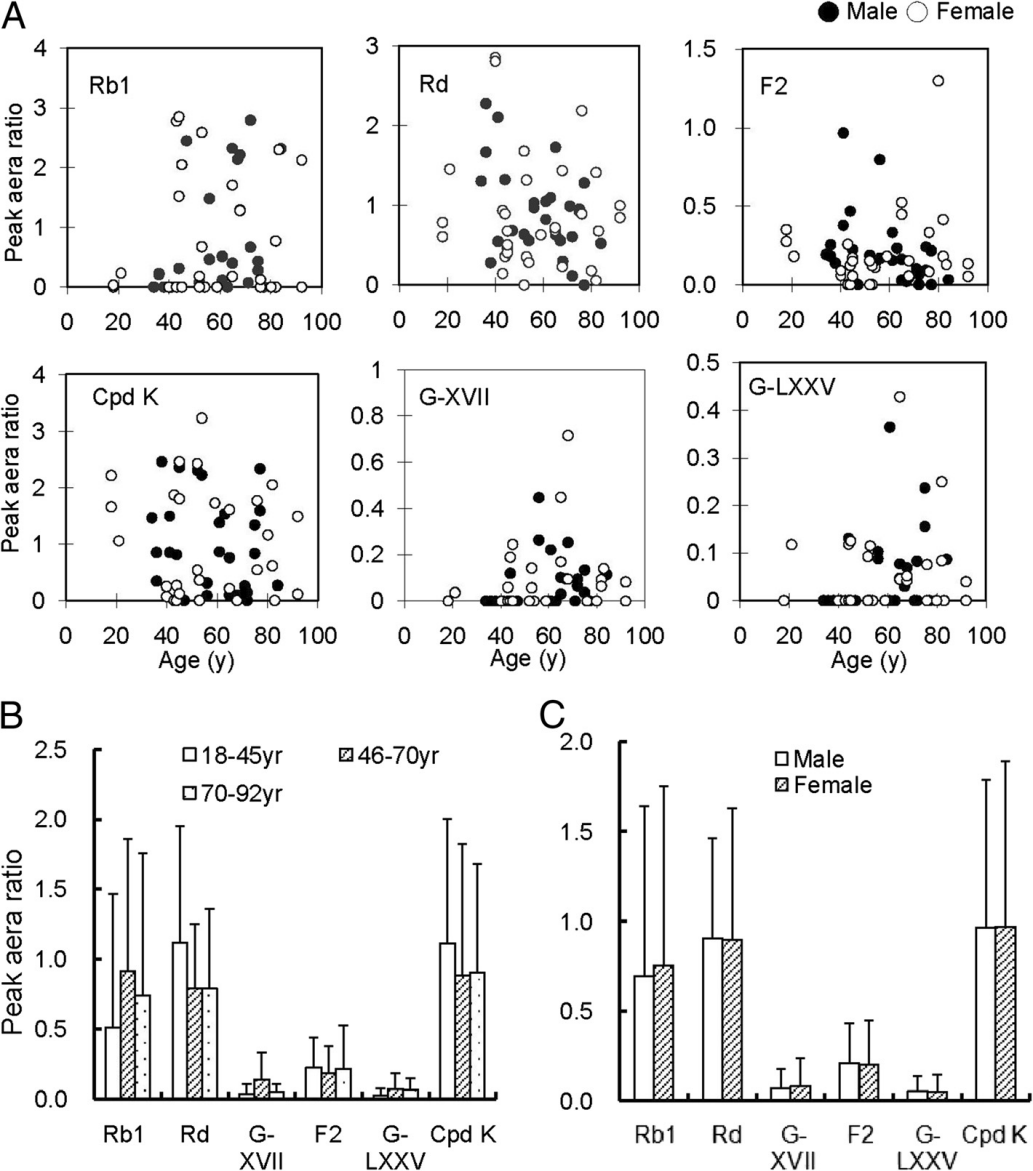
Ginsenoside Rb1 metabolism and its metabolite formation by gut bacteria samples from 58 healthy individuals (A) of different ages (B) and sexes (C).

When Rb1 was separately incubated with gut bacterial samples from 58 individuals aged 18-92 years (30 females and 28 males; range, 39.6-75.3 years), great inter-individual variations were observed in terms of the remaining Rb1 and the amounts of metabolites formed *via* the Rd and gypenoside pathways (Figure 3A). However, when compared by age, there were no significant differences in Rb1 elimination and each pathway among individuals aged 18-45 years (20 individuals; range, 29.2-47 years), 46-70 years (22 individuals; range, 52.7-65.9 years), and 71-92 years (16 individuals; range, 72.6-85.4 years) (Figure 3B). No sex differences (*P* values: 0.3 ~ 0.5) were observed in Rb1 metabolism and each pathway by human gut bacteria (Figure 3C).

### Degradation of Rb1 and Rd in SGF

The calibration curves of Rb1, Rd, G-XVII, F2, Rg3, Cpd K, and Rh2 in SGF showed good linearity (*r*^*2*^ > 0.998, *P* < 0.001) over the concentration ranges tested (Table 2).

**Figure 4:**
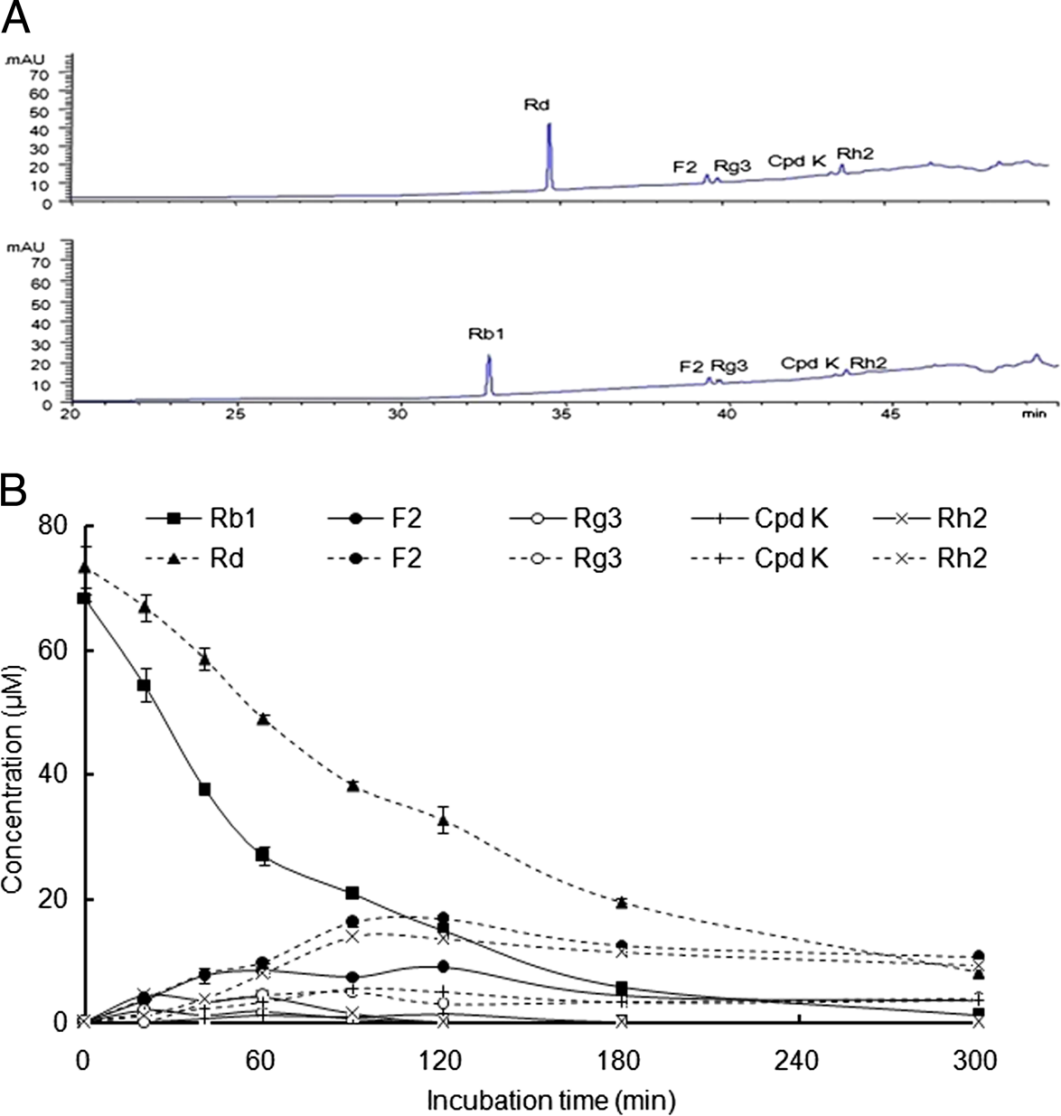
HPLC-UV chromatograms (A) and degradation time-courses (B) of Rb1 (solid line) and Rd (dotted line) in SGF.

Rb1 was rapidly degraded under acidic environments to half of its original amount in 1 h, with <10% remaining at 3 h (Figure 4). Ginsenosides F2, Rg3, Cpd K, and Rh2 were all detected in SGF. However, Rd was not detected throughout the incubation period (Figure 4A). When Rd was incubated in SGF, it was rapidly degraded to half its initial amount in 2 h. The same products (F2, Rg3, Cpd K, and Rh2) were generated. In both cases, F2 was the most abundant product in SGF, and the gypenosides were not detected (Figure 4A).

**Figure 5:**
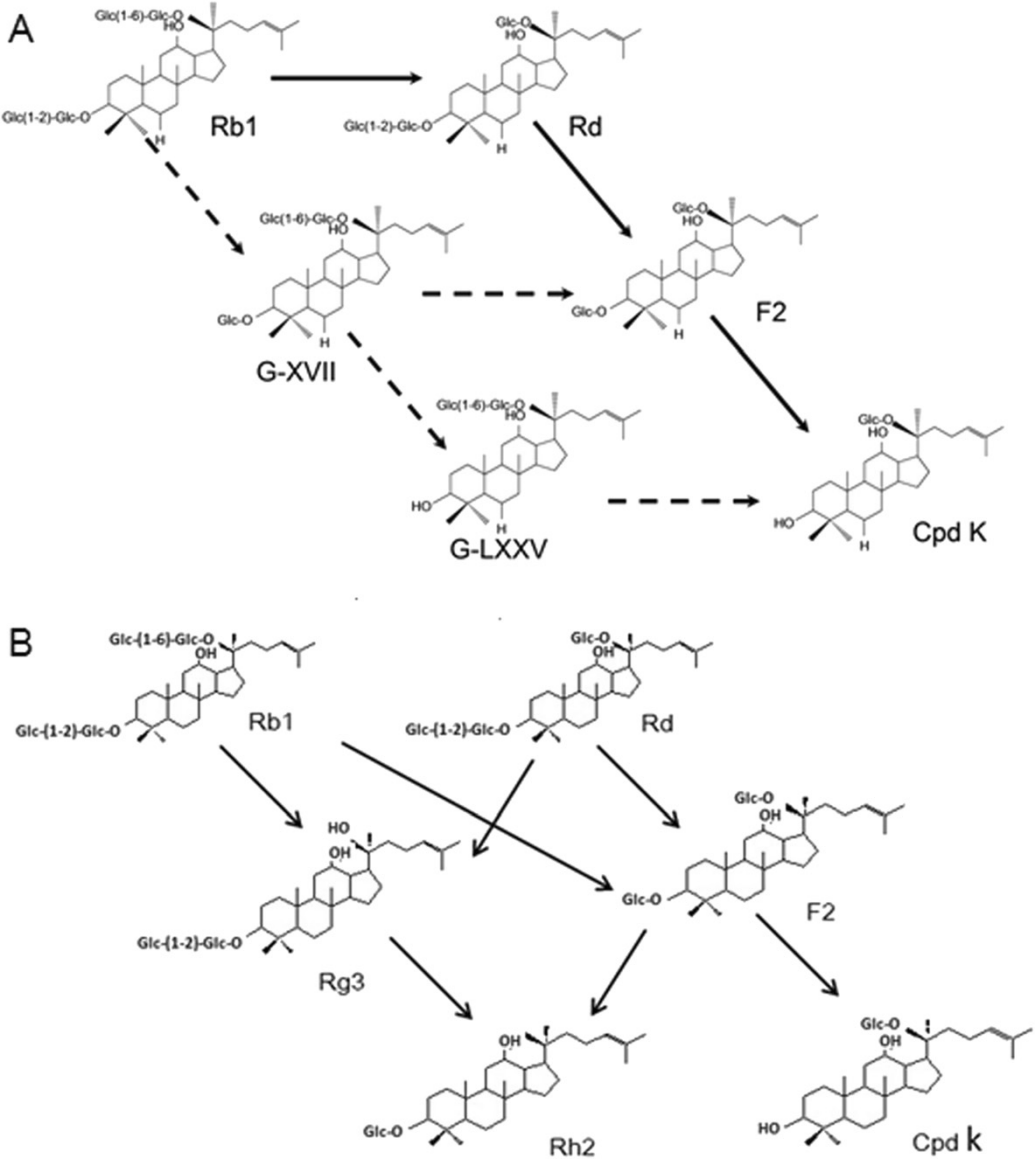
**Pathways of (A) Rb1 biotransformation by human intestinal microflora (→ major pathway;** ⤏ **minor pathway) and (B) Rb1 degradation in SGF.**

The *in vitro* bacterial biotransformation experiments suggested the existence of metabolic pathways for Rb1 in human gut bacteria as follows: Rb1 lost one C-20 glucose to generate Rd (major) and one C-3 glucose to generate G-XVII (minor); Rd lost another C-20 glucose to generate F2 and then one C-3 glucose to generate Cpd K; G-XVII further lost one C-20 glucose to form F2 (major), one C-3 glucose to generate G-LXXV (minor), or both; both were finally metabolized to Cpd K. However, under acidic conditions, the degradation of Rb1 was initiated by simultaneous loss of two glucose molecules from both the C-3 and C-20 positions to generate F2 (major) or Rg3 (minor). The metabolic pathway of Rb1 in human gut bacteria shown in Figure 5A and the degradation pathways of Rb1 and Rd in SGF shown in Figure 5B were proposed for comparison.

## Discussion

Ginsenosides generally have poor oral bioavailability because of their low membrane permeability, active biliary excretion, biotransformation, and so on [12]. Their deglycosylated products formed by bacterial conversion in the gut lumen are more permeable and were demonstrated to be bioactive [36]. As one of the main PPD-type ginsenosides, ginsenoside Rb1 was deglycosylated *via* Rb1 → Rd → F2 → Cpd K (Rd pathway) [26,37]. Another pathway *via* Rb1 → G-XVII → G-LXXV/F2 → Cpd K has been reported in rats [24], single fungal strains [20-22], and bacteria such as *Fusobacterium* sp. and *Bifidobacterium longum* sp. [38,39].

In this study with application of both LC-MS and LC-DAD techniques, G-XVII and three metabolites (Rd, F2, and Cpd K) were identified from the Rd pathway and G-LXXV was tentatively assigned. The existence of both the Rd and gypenoside pathways of Rb1 metabolism in human gut bacteria was confirmed. The conversion of Rb1 to Cpd K *via* both G-XVII → G-LXXV and G-XVII → F2 was also evidenced, for the first time, by the observation that G-XVII produced G-LXXV, F2, and Cpd K, while Rd only generated F2 and Cpd K, in human gut bacteria *in vitro*.

The gypenoside pathway was demonstrated to be the minor pathway of Rb1 biotransformation in human gut bacteria by comparing the maximum levels of Rd and F2 with those of their respective positional isomers G-XVII and G-LXXV determined from a time-course study. Moreover, the maximum levels of the gypenoside products were reached more rapidly than the products of the Rd pathway, indicating that the gypenoside pathway was a transient intermediate process. In contrast, the conversion rate of Rd to Cpd K was faster than that of G-XVII to Cpd K, as judged by the times required to reach the maximum level of Cpd K when Rd and G-XVII were separately incubated with human gut bacteria. These findings suggested that the removal of the remote glucose molecule at the C-20 position by human gut bacteria was faster than that of the glucose molecule from the C-3 position, while further cleavage of the second glucose from the C-3 position is a much more difficult process, leading to the formation of F2 much more rapidly than the formation of G-LXXV from G-XVII. *Eubacterium sp*., *Streptococcus sp.*, and *Bifidobacterium sp*., which were more potent in hydrolyzing gentiobiose than sophorose, metabolized Rb1 to Cpd K *via* Rd rather than G-XVII [39]. However, *Fusobacterium K-60*, which hydrolyzed sophorose more potently than gentiobiose, metabolized Rb1 to Cpd K *via* G-XVII. Bifidobacteria are probiotics and belong to the predominant gut bacteria, while streptococci and fusobacteria are pathogenic bacteria and are in the minority in healthy humans [40]. This may explain why the Rd pathway was the major pathway, while the gypenoside pathway was the minor pathway of Rb1 in human gut bacteria from healthy volunteers.

In this study, rapid degradation of Rb1 was observed in SGF and its products including F2, Rg3, Cpd K, and Rh2 were detected, consistent with the findings obtained from previous *in vitro *[41] and *in vivo *[42] studies. Rd yielded the same four products in SGF. However, gypenosides were not detected in SGF in both cases, indicating that when Rb1 is taken orally, gypenosides may only be formed through bacterial biotransformation in the gut lumen.

Great inter-individual variations in Rb1 metabolism by human gut bacteria were observed with both the gypenoside and Rd pathways owing to the great individual variations in the species and amounts of the gut bacteria. These variations showed no differences between sexes and ages.

Thus far, only the Rd pathway has been reported *in vivo* in the human body [25,26]. The minor amount of gypenosides formed by the human gut bacteria may provide beneficial effects for the human body. Thus, further studies are warranted to evaluate the contributions of these gypenosides to the health-promoting effects of ginsenoside Rb1 *in vivo*.

## Conclusions

Rb1 was metabolized to G-XVII, F2 (major) or G-LXXL (minor), and finally Cpd K by human gut bacteria *in vitro*.

## Abbreviations

DMSO: Dimethyl sulfoxide; PBS: Phosphate-buffered saline; PPD: 20(*S*)-protopanaxadiol; G-XVII: Gypenoside XVII; G-LXXV: Gypenoside LXXV; Cpd K: Compound K; HPLC-UV: High-performance liquid chromatography with a UV detector; MS: Mass spectrometry; ESI: Electrospray ionization; BHI: Brain heart infusion; SGF: Simulated gastric fluid; QC: Quality control; DAD: Diode array detector; LC/MSD: Liquid chromatograph/mass selective detector; IS: Internal standard; SD: Standard deviation.

## Competing interests

The authors declare that they have no competing interests.

## Authors’ contributions

RY conceived the study. JPCL, YTW, and RY designed the study. WIL carried out the ginsenoside Rb1 biotransformation by pooled and individual human gut bacteria. HS carried out the experiments on metabolism by human gut bacteria and acidic stability. JQR collected the human fecal samples and prepared bacterial suspensions. SLL and JPCL performed the statistical analysis. WIL, HS, and RY wrote the manuscript. All authors read and approved the final manuscript.

## Supplementary Material

Additional file 1: Table S1 Intraday and interday variability for the assays of ginsenoside Rb1, Rd, F2 and compound Kin incubates with human gut bacteria. Click here for file
