# Supplementary material for: Biotransformation of ginsenoside Rb1 via the gypenoside pathway by human gut bacteria
Source: Chin Med. 2013 Nov 23;8:22. doi: 10.1186/1749-8546-8-22 (PMC4175505; doi:10.1186/1749-8546-8-22)
Supplement: Additional file 1: Table S1 — Intraday and interday variability for the assays of ginsenoside Rb1, Rd, F2 and compound Kin incubates with human gut bacteria. [file 1749-8546-8-22-S1.docx]

Additional file 1: Table S1. Intraday and interday variability for the assays of ginsenoside Rb1, Rd, F2 and compound Kin incubates with human gut bacteria

| Sample | Concentration Spiked (mM) | Intraday (n = 3) | | | Interday (n = 3) | | |
| --- | --- | --- | --- | --- | --- | --- | --- |
|  |  | Detected (mM) | RSD (%)* | Recovery (%)† | Detected (mM) | RSD (%)* | Recovery (%)† |
| Rb1 | 0.05 | 0.0420 ±0.0019 | 4.54 | 84.04 | 0.0429 ±0.0024 | 5.56 | 85.81 |
|  | 0.2 | 0.2004 ±0.0013 | 0.66 | 99.81 | 0.2027 ±0.0028 | 1.40 | 98.67 |
|  | 0.8 | 0.7789 ±0.0045 | 0.58 | 97.36 | 0.7792 ±0.0054 | 0.69 | 97.40 |
| Rd | 0.03125 | 0.0305 ±0.0003 | 0.94 | 97.47 | 0.0308 ±0.0002 | 0.63 | 98.65 |
|  | 0.125 | 0.1222 ±0.0020 | 1.65 | 97.75 | 0.1235 ±0.0021 | 1.73 | 98.77 |
|  | 0.5 | 0.4872 ±0.0034 | 0.70 | 97.44 | 0.4871 ±0.0111 | 2.29 | 97.41 |
| F2 | 0.01625 | 0.0154 ±0.0001 | 0.71 | 95.05 | 0.0155 ±0.0003 | 2.24 | 95.52 |
|  | 0.065 | 0.0651 ±0.0014 | 2.19 | 99.78 | 0.0658 ±0.0020 | 3.00 | 98.70 |
|  | 0.26 | 0.2575 ±0.0003 | 0.11 | 99.02 | 0.2624 ±0.0044 | 1.66 | 99.07 |
| Compound K | 0.0103 | 0.0061± 0.0003 | 4.42 | 59.69 | 0.0074 ± 0.0010 | 13.74 | 72.23 |
|  | 0.0413 | 0.0389± 0.0002 | 0.53 | 94.36 | 0.0392 ± 0.0005 | 1.22 | 95.06 |
|  | 0.165 | 0.1454± 0.0026 | 1.71 | 111.87 | 0.1510 ± 0.0102 | 6.76 | 108.48 |
| *RSD (%) = (standard deviation/mean) × 100. | | | | | | | |
| †Recovery (%) = [1 - \|mean concentration measured - concentration spiked\|/concentration spiked] × 100. | | | | | | | |
